# “It doesn’t feel like exercise”: a realist process evaluation of factors that support long-term attendance at dance classes designed for healthy ageing

**DOI:** 10.3389/fpubh.2023.1284272

**Published:** 2023-12-20

**Authors:** Abby Haynes, Anne Tiedemann, Gail Hewton, Julie Chenery, Catherine Sherrington, Dafna Merom, Heidi Gilchrist

**Affiliations:** ^1^Sydney Musculoskeletal Health, Institute for Musculoskeletal Health, University of Sydney and Sydney Local Health District, Sydney, NSW, Australia; ^2^Sydney School of Public Health, University of Sydney, Sydney, NSW, Australia; ^3^Gold Moves Australia and RIPE Dance, Noosa, QLD, Australia; ^4^Gold Moves Australia, Moreton Bay, QLD, Australia; ^5^School of Health Sciences, Western Sydney University, Penrith, NSW, Australia

**Keywords:** physical activity, fall prevention, older people, music, program adherence, mechanism

## Abstract

**Introduction:**

Dance can positively impact older people’s health and wellbeing across cultures and socioeconomic groups, countering age-related physical, sensorimotor and cognitive decline.

**Background/objectives:**

The RIPE (Really Is Possible for Everyone) Dance program aims to improve older people’s physical, mental, cognitive and social wellbeing by integrating engaging dance sequences with evidence-based fall prevention exercises. We sought to identify what mechanisms support observed long-term participation in this program, including by people living with challenging health conditions.

**Methods:**

Following a realist evaluation approach, we co-developed and tested program theories iteratively with participant interviewees (*n* = 20), dance teachers (*n* = 2) and via observation of a dance class. Initial data were dual-coded and emergent findings were interrogated by the research team. Findings were organised to express *Program activities + Context + Mechanism = Process outcomes* configurations.

**Results:**

We identified four program theories comprising 14 mechanisms which explained long-term attendance: 1. RIPE Dance benefits my body and mind (trust in the program, belief in health benefits), 2. RIPE Dance helps me feel good about myself (self-efficacy, pride in achievement, psychological safety, defying expectations, feeling valued), 3. RIPE Dance creates camaraderie (social connection, mutual support, rapport with the teacher), and 4. RIPE Dance is uplifting (raised spirits, fun, synchrony, musical reactivity).

**Conclusion:**

The RIPE Dance program provides effective and enjoyable ‘exercise in disguise’ for older people with diverse mobility profiles.

**Significance/implications:**

This research confirms that participation in dance can contribute significantly to healthy, happy ageing. Findings detail program activities that were most strongly associated with process outcomes, offering guidance for further program development, implementation and scaling up.

## 1 Introduction

Dance has the potential to be an effective, inexpensive, scalable and sustainable community-based activity that contributes significantly to healthy, active ageing (1–4). The unique combination of aerobic and anaerobic exercise with mind–body practices enables dance to target multiple features of ageing simultaneously (3). Dance can positively impact people’s physical, mental, cognitive and social health and wellbeing across cultures, age and socioeconomic groups (4), and may have particular value countering age-related physical, sensorimotor and cognitive decline (5). Meta-analysis indicates that structured dance can be a safe and beneficial form of exercise which is as effective, or more effective, for improving a range of health outcomes than other forms of structured exercise (6).

Dance is increasingly used to prevent and/or manage certain health conditions. For example, a dance program for women following breast cancer treatment provided improvements in anthropometric measures, fitness levels and psychosocial wellbeing (7). In particular, dance has emerged as an important tool in the treatment of Parkinson’s Disease (8) and dementia (9) (including Alzheimer’s disease (10)), and is thought to have benefits for people with autism spectrum disorder (11), stroke (12) and conditions such as rheumatoid arthritis, fibromyalgia (13) and chronic pulmonary disease (14).

### 1.1 Dance and fall prevention

Direct evidence of the effect of dance on fall prevention is still needed, but there is increasing evidence that dance interventions can reduce the risk of falls (15–17) by targeting risk factors such as impaired balance, postural control, gait, agility, leg muscle weakness, aerobic power, lower body muscle endurance and cognitive impairment (18–22). Dance may have other neurological benefits too. A study comparing older adults who danced or took part in aerobics classes found an increase in the volume of the hippocampus in both groups, but in the dancers this appeared to be linked with improved balance (23).

### 1.2 Dance and psychological health

The positive psychological impacts of dance for older people are well-established (24), including for those with cognitive impairment (2). Meta-analyses indicate that participants in dance interventions show significantly fewer depressive symptoms than controls (25). These impacts have been found in diverse populations and settings. For example, a dance program for older people in an acute hospital showed significant improvements in mood, sleep, relaxation, creativity and friendly interaction with other patients (26). Even online dance classes can have positive impacts on mood and self-esteem (27).

There is increasing interest in the elements of dance that produce these effects. The role of music is thought to be key. Campion and Levita (28) found that both dancing and listening to music passively enhanced positive affect, decreased negative affect and reduced feelings of fatigue. However, a study of psychiatric patients with depression found that those who participated in dance showed significantly less depression than comparisons who only listened to the music (29). This suggests that music is one of several elements that contribute to positive psychological impacts of dance. Another is embodiment: attentive experience of the lived body involving mindful sensory and kinaesthetic awareness that connects body and mind (9). Mind/body experiences can enhance engagement with physical activity and have profound impacts on wellbeing (30). As Coaten argues, *“The extent to which one is grounded in the lived-body experience affects one’s perceptions, understandings and sense of relatedness to the world”*[9:677]. This may partly explain the emotional impact of expression through dance movement (11, 31) which can enhance feelings of joy, energy, strength (29), empowerment, pride and determination (32). The interoceptive accuracy associated with heightened body awareness through dance may also positively impact wellbeing, empathy, altruism, emotional resilience and efficient decision-making (33). A third element is synchrony: the pleasure of moving in unison with others. Synchrony appears to affect physiological responses and cortical activity (34), and can generate a group state of positive physicality, emotion and arousal which has been described as *“collective effervescence”* (35).

### 1.3 Dance and social bonding

A growing body of literature indicates that synchrony strengthens social attachment among group members (11, 36, 37) and can foster cooperation, resolve conflict (38, 39), and increase intercultural empathy (40). Studies of synchronous non-dance physical activity have also shown positive impacts on group bonding (41), but the incorporation of music and harmonious expressive movement through dance is likely to elevate this effect (38). Social bonding may be enhanced by shared exertion (42) (such as in a physically challenging dance class) and by neural stimulation of social observation networks (11). Social connectedness and a sense of belonging predicts increases in self-esteem (43) and other positive mental states, partly because it releases endorphins which are associated with feelings of pleasure, gratification and trust (36).

### 1.4 Dance and neuropsychological/cognitive health

Dance is becoming established as a tool for targeting age-related cognitive decline, as well as sensorimotor and physical decline (5). Structured dance demands learning and recall of complex routines and musical reactivity which combine physical activity with sensory, motor, cognitive, social, emotional, rhythmic and creative neurobehavioral processes (11, 38). This creates a ‘neural synchrony’ linked with cognitive processing of time, sound and experience (11). Studies show that dance has more significant cognitive benefits for older people than physical activity alone (44). For example, randomised trials have found dance interventions to have greater positive impact on cognitive function among healthy older adults than an aerobic program (1), and greater reduction of dementia risk factors compared to a physical activity program (45). A study of dance-training for older people found a significant increase in the para-hippocampus (a region of the brain involved in learning and memory) in the dance group compared to controls who were participating in a sports intervention (23, 46).

For people with early dementia or elevated risk of dementia, dance has been shown to be safe with high levels of acceptability and positive neurological impacts across different cultural and disadvantaged groups (47–50). Reported impacts include improvements in learning skills, immediate and delayed recall, attention and social functioning (15, 51). Wider cognitive benefits of dance have also been identified, including stimulation of creativity (11, 52).

People with Parkinson’s Disease may especially benefit from the cognitive impacts of dance. Reviews indicate that, compared to other types of exercise, dance improves symptoms and outcomes in Parkinson’s patients, especially motor symptoms, with positive effects on balance, gait, functional mobility, cognition and wellbeing (53, 54). Positive effects may be supported by internalized musical rhythm affecting gait (55), and ‘action representation’ (observing and mirroring others’ movements) which is a feature of teacher-led dance class participation (56).

### 1.5 Sustaining older people’s participation in community-based physical activity

Despite clear evidence of the benefits of physical activity generally (57), adherence to community-based physical activity programs (including those which focus on fall prevention (58)) remains a major challenge (59) with approximately 50% of participants dropping out within 6 months (60). Adherence rates may be considerably higher for older people with estimates between 69 and 75%, but this figure probably reflects lower initial uptake, particularly by those who are least active (61, 62). Non-adherence by older people in exercise programs is associated with pre-existing poorer health and physical and cognitive ability, lower socio-economic status, and higher levels of depression and perceived risk of falling (63). Thus those who are in most need experience compounded disadvantage. As Stathi et al. argue, *“The greatest challenge facing activity promoters lies with finding strategies that can help attract older adults to activity initiatives and keep them attending and wanting more”* [64:7].

Adherence of older people to physical activity programs is associated with individual factors such as lifelong history of physical activity, intrinsic motivation, self-efficacy; facilitator characteristics (particularly their personality, professionalism and ‘humanised’ approach); program design (including location, affordability, the use of music and tailored content); environmental considerations such as accessible equipment and welcoming facilities; social features which support a sense of belonging; and perceived physical and psycho-social benefits (60, 62, 64). Given that dance ticks many of these boxes, it is, perhaps not surprising that participation in dance interventions may have relatively low attrition (22). This may be enhanced by mechanisms of engagement which are particularly associated with dance such as emotional expression, connection with music and social interaction (65). As Franco et al. argue, studies show that many older people consider dance to be a stimulating and joyful activity which also provides opportunity for socialisation – a far cry from the tedium, isolation and deficit-orientation of some traditional exercise and fall prevention programs (19, 66).

### 1.6 The program being evaluated: ripe dance

The RIPE (Really Is Possible for Everyone) Dance program offers classes for people aged 55+ with three different activity levels catering for people with high physical function, moderate physical function and impaired mobility and balance (67). The RIPE approach integrates engaging, and often playful, creative dance sequences and routines with strategies for promoting cognitive stimulation, mental health, social connectedness and evidence-based exercises for fall prevention and for general strength and mobility. Class structure and dance content are based on contemporary and jazz dance techniques incorporating other dances genres, making the classes stylistically eclectic. All classes are 75 min duration. Those for people with moderate to high physical function are all standing, while classes for people with mobility and balance issues begin seated and progress to standing unassisted or assisted using aides such as chairs, walkers or walking sticks. There is also option to remain seated for the entire session. While dance content across classes may be similar in movement action and style, the levels of activity differ in physical and cognitive demand, choreographic complexity and movement/music tempo.

The RIPE Dance program is founded on an approach that is specially designed to maintain or improve health, mobility and wellbeing in older people with a range of physical activity, mobility and health profiles. It aims to enable everyone to experience the joy and benefits of dance in a fun, safe, supportive and welcoming environment, including those who believe they cannot dance. The designers and teachers are experienced in dance, choreography, adult education and community engagement. They deliver classes using a relational pedagogical model characterised by respect, empathy, authenticity and social learning (68, 69). The RIPE Dance approach is used in classes by teachers of RIPE Dance and Come Dance community dance practices and is promoted by Gold Moves Australia. In this paper we will use the term RIPE Dance to include all community dance classes that use the RIPE Dance approach, including Come Dance classes.

Many participants in these classes have attended for several years, and over 10 years in some cases. They include men and women, people in their 90s, and people with conditions such as cancer, obstructive pulmonary disease, Parkinson’s disease, osteoporosis, anxiety and depression. This study sought to better understand what appeals to participants about these classes and supports their long-term attendance, including the role of the dance teachers and their pedagogical approach – a current gap in the literature (70). Specifically, we sought to identify what is working for participants, under which conditions and why.

## 2 Methods

### 2.1 Methodology: realist process evaluation

Process evaluation investigates a program’s implementation, function and contextual interactions in order to explain (insofar as this is possible) how and why it generates outcomes (71). Process evaluation does not determine whether final program outcomes are achieved, but it can identify proximal outcomes, i.e., process effects such as acceptability and engagement, that make achieving the program’s distal outcomes more likely (72). This study combined the focus of process evaluation on understanding proximal outcomes with a realist evaluation methodology (73).

Realist evaluation is a form of theory-driven evaluation which develops and tests theories about how a program is supposed to work (74). It uses purposive sampling and pragmatic data collection to follow rich veins of information to identify the most plausible explanations for how program activities work in different circumstances. This results in causal explanations that combine information about contextual features, underlying mechanisms and program outcomes (74, 75). While these explanations are acknowledged to be a social construct that cannot offer definitive answers, the experiences and views that inform them are real (76). This means that realist evaluation can generate robust and transferable knowledge about how a program is most *likely* to work which can be further tested and refined in subsequent studies (76).

### 2.2 Initial program theory development

The initial program theory was developed at a workshop with the research partnership team which comprised two dance teachers who designed and deliver the RIPE Dance program, and the academic researchers leading this study. Rough ideas about what worked for dance class attendees were brainstormed, informed by the teachers who had honed an informal program theory over years of delivering classes, and supplemented with ideas from the researchers derived from their expertise in physical activity and healthy ageing. Notes from the workshop were written up as draft theories that were further refined via several iterations of email until the team agreed they were a sound reflection of our working hypotheses and were ready to be tested in interviews. During this phase, and throughout the data collection and analysis, AH and HG were scanning the literature to learn from other studies and extant theories which might inform this work.

### 2.3 Recruitment

Participants of six RIPE Dance classes were invited by their class teacher to complete a survey to evaluate the RIPE Dance program. Surveys were distributed in December 2022 and followed up at the start of term in February 2023. Seventy-seven were distributed and 62 were returned (81% response rate). We recruited interview participants from respondents who ticked a box saying they gave permission for the researchers to invite them to take part in an interview (*n* = 51). We sent three rounds of invitations (*n* = 29 invitees, 10 + 10 + 9) via email which contained an embedded consent form and included the participant information statement as an attachment.

We sampled purposively for maximum variation in age, self-rated level of mobility, past experience with dance, number of close friends and reported physical and mental health challenges. We also aimed to speak to at least two people from each of the six classes, some people with carer roles and, ideally, to the only two current male class members.

Recruitment ceased when: a. All participants had had sufficient time to complete and return their surveys (thus ensuring we had the fullest sample frame), b. We had achieved good coverage across the purposive criteria described above, and c. Interviewees consistently described the key features of our evolving program theory unprompted and then confirmed the formal theory without suggesting any additional concepts.

### 2.4 Data collection

In realist evaluation, interviews and observations are used as tools for testing and refining theories (77). The interview guide was developed to prompt interviewees to reflect on views that we believed were likely to inform our theories: reasons for enrolling in the classes, and reasons for adherence, including any impacts on health, wellbeing, identity and social engagement (Supplementary material S1). Following the realist interview approach (78), questions explored perceptions about process outcomes, underlying mechanisms and any relevant contextual information. Interviewees were asked explicitly about our current program theories in the latter part of the interview. This meant that early interviewees commented on the initial program theory, while later interviewees commented on more refined versions of the theory.

Interviews were conducted by the same, experienced, qualitative researcher (AH) via telephone or teleconferencing software, depending on each interviewee’s choice. All interviews were audio recorded and transcribed verbatim by a professional transcription service. Transcripts were corrected by the interviewer and uploaded to NVivo 1.7.1 for data management and coding.

Following these interviews and refinement of the program theories, two qualitative researchers (AH and HG) independently observed a RIPE Dance class via videolink set up by the teacher. The class selected was one targeted at less agile older people so we could observe strategies for engaging people with pronounced mobility constraints in enjoyable dance. An observation guide was used which focused on looking for observable information that might contradict, support or refine the program theories and their mechanisms (Supplementary material S2).

### 2.5 Data analysis

A thematic coding approach was used based on the initial program theories (which were modified extensively during early analysis) with double coding to identify specific elements within these theories: program activities, contextual features, possible causal mechanisms and any process outcomes relating to engagement in the dance classes. Two researchers (AH and HG) trialled this approach with early transcripts and AH coded subsequent data.

An analytic memo and a table of evolving program theories and possible mechanisms was maintained and updated by AH following interviews. These documents collated notes about the current theories being tested and any revisions made, and were discussed with HG after every few interviews. Following the 10th interview, the initial and revised program theories were presented with corroborating data to the research partnership team for discussion. Queries and reflections from the memo were also shared and discussed. The team negotiated to merge two theories, rename one theory, remove several mechanisms and add a new mechanism. Subsequent observational data was discussed by the two observers in relation to these theories. This resulted in additions to the program activities and minor refinements of the language used to describe two mechanisms.

### 2.6 Linking the program theory components

Realist evaluation results are typically presented in Context+Mechanism = Outcome configurations (74). This structures findings into propositions which describe the most likely causal pathways identified in the research (79). Others have modified this framework to include program or intervention activities resulting in Intervention+Context+Mechanism = Outcome or Strategy+Context+Mechanism = Outcome configurations (80–82). We favoured this approach because it details the program activities that are most strongly associated with process outcomes and identifies how they seem to be linked. This provides clearer information about the ‘essential ingredients’ that can guide further program development, implementation and scaling up. Given that this was a process evaluation of a community-based program, we opted for Program activities+Context+Mechanism = Process outcomes as the most accessible phrasing for readers without research backgrounds. This configuration was used as a template to describe our findings, and discuss them amongst the research partnership team. We also developed a simple figure to illustrate the relationships we believed existed between the program theories, based on participants explanations of ‘how dance classes work’.

Ethical approval was provided by the University of Sydney’s Human Research Ethics Committee, study reference 2022/772. All participants gave written or emailed informed consent to take part in interviews and be observed in class, and for their deidentified data to be used in publications. Interviewees were asked to verbally reconfirm their willingness to take part in an interview at the start of the interview.

## 3 Results

A total of 20 dance class participants took part in an interview, 18 female and 2 male. They were aged between 66 and 92, with an average age of 78. There were at least two interviewees from each of the six RIPE Dance classes. Attendance at these classes ranged from approximately 8 months to over 10 years. The interviewees all lived in the Sunshine Coast or Moreton Bay regions of Queensland, Australia. As determined by postcodes, 12 lived in high socioeconomic areas, six in mid-range and two in low socioeconomic areas. All but one interviewee reported a health condition, many of which were potentially mobility-challenging; for example, 11 reported osteoarthritis, six reported osteoporosis, and three reported Parkinson’s Disease. Other reported conditions included angina, cancer, gout, stroke, hypertension, diabetes, visual and hearing impairments and depression. Six reported they had fallen at least once in the previous 12 months, but none had sustained an injury. Interviews lasted between 36 and 56 min, with an average duration of 48 min. Observation of the dance class for least agile participants lasted for 90 min in order to observe pre-and post-class dynamics.

The data analysis produced four program theories: 1. RIPE Dance benefits my body and mind, 2. RIPE Dance helps me feel good about myself, 3. RIPE Dance creates camaraderie, and 4. RIPE Dance is uplifting. Within these program theories we identified 14 mechanisms which were strongly supported by interviewees as providing the best explanations for their long-term and enthusiastic attendance at RIPE Dance classes.

While these results are presented below as four discrete program theories, we note that they are closely related and work together to effect engagement with the RIPE Dance program. This means that tidy segregation of mechanisms in the data tables that follow may not reflect the experiential reality of interviewees who generally described synergistic impacts of multiple features of the classes generating a cluster of responses. For example, this interviewee is alluding to all four theories in her explanation of engagement with dance classes:

… the instruction is very clear, the environment is quite safe and it’s also very non-judgmental. You make a fool of yourself but nobody bothers because everybody else is doing the same thing. So we like it for that and the social contact, and it’s a very light-hearted session, so we really come out feeling good. And the sessions she arranges for us are amazing I think…. mainly I think in the challenge. It’s a neurological challenge. As I said, coordinating your arms and legs, and using all your joints and muscles to rise up and down on your feet, and you stretch your legs and sit-up [tall], do sit-to-stands. It’s exercise, but she’s actually trying to work all parts of the body in the session. And each term is a different set of dance steps … so we don’t get too used to them, you always have to learn new ones (female participant, 83 years).

**Figure 1:**
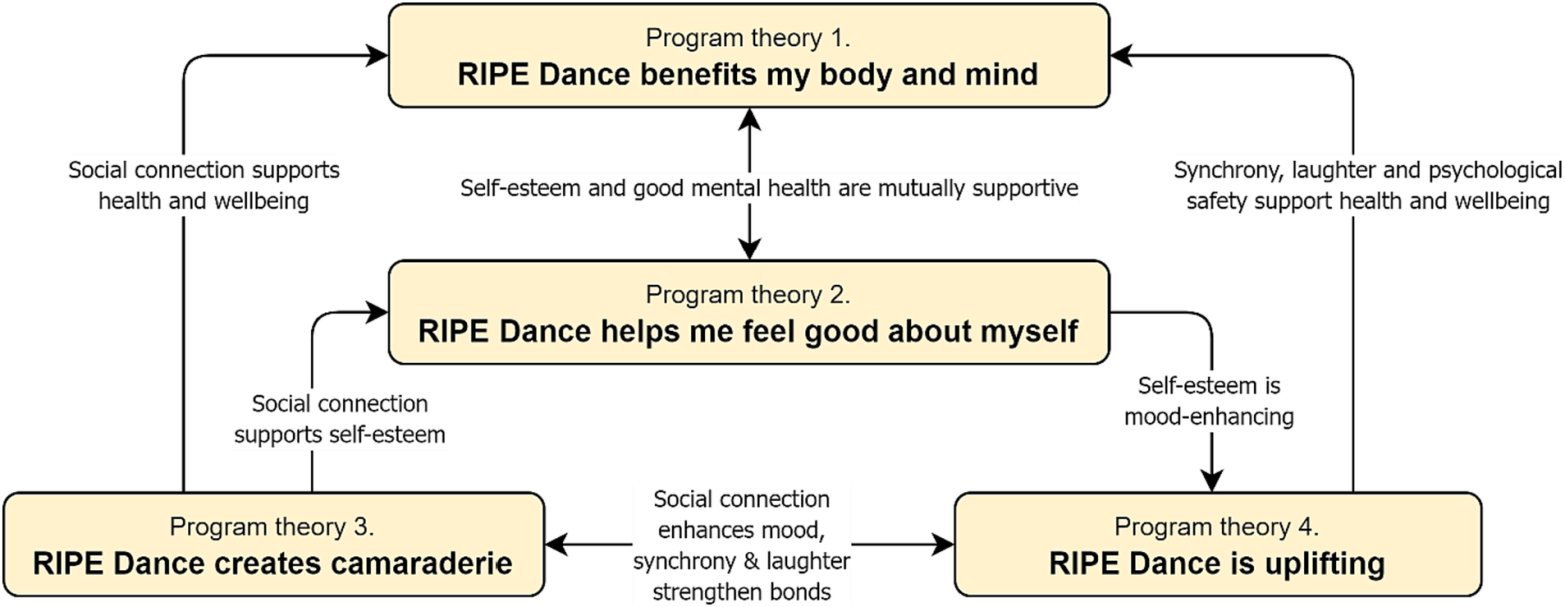
Suggested relationships between program theories in the analysis.

The suggested relationships between the four program theories identified in our analysis (Figure 1) indicate that some are mutually supportive, while others are unidirectional. Table 1 provides a more detailed overview of each program theory.

**Table 1:** Overview of the program theories developed to explain the process outcome of engagement with RIPE Dance classes.

Program theory	Program activities	Context	Mechanisms
Theory 1: RIPE Dance benefits my body and mind	RIPE dance classes are promoted as supporting healthy ageing and fall prevention Classes incorporate a wide range of evidence-based exercises for strength, mobility and fall prevention (e.g., standing balance challenges, sit-to-stand repetitions, knee extensions) Each class incorporates sequences of progressively taxing movement with a warm up and cool down Classes are delivered by highly skilled dance teachers with extensive gerontological knowledge Participants provide information about their health and are asked to advise teachers of any changes to aid responsive instruction (teachers check in with participants before and during class) Teachers provide in-class education about the aims and benefits of different exercise sequences Teachers frequently remind participants to work at own level with individual modifications for those with specific conditions Tailored use of chairs and cushions for seated movement and standing balance support where appropriate Teachers ask participants to remember and call out what steps come next (building on the cognitive challenge)	Ageing is associated with physical, cognitive and psychological decline, and increased risk of falls (83) Physical activity plays a vital role in countering this decline (84) but too few older people are sufficiently physically active (a situation exacerbated by COVID-19 (85)) (86) Exercise can feel daunting and unsafe for older people (87) who will not participate unless they perceive it as acceptable and beneficial (88)	Trust that the RIPE approach ‘is right for me’: belief that it is safe, age appropriate and is individually tailored for each person’s needs (89) Belief in and/or experience of health benefits (physical, psychological and cognitive) from participating in classes including perceived improvements in strength, balance, flexibility, mobility, posture, physical literacy and/or sleep; stress reduction; amelioration of grief and a stronger commitment to physical activity in general (61, 63, 90)
Theory 2: RIPE Dance helps me feel good about myself	A repertoire of new sequences each term designed to provide challenge while also building on previously mastered steps Repetition of movements to support learning and building confidence balanced with variety of new movement to maintain interest and motivation Highly responsive teaching: continually modifying movements and routines for individuals and the group to create achievable challenge A ‘run through’ of all moves before each sequence starts in the learning stages and subsequently as required Clear, continuous instructions with movements modelled by the teacher during each sequence Positive feedback and celebration of group and individual successes (e.g., the group bow and applaud themselves at class end) Teacher models empathy, respect and acceptance Explicit reiterated emphasis on having fun and not worrying about mistakes *Ad hoc* prizes awarded that recognise attendance rates, resilience and positive group interactions	Lack of confidence in one’s capabilities is a major barrier to motivation for physical activity Adherence to exercise is associated with supportive facilitation and environments (59, 62, 91) Many older people experience ageism, including negative stereotypes that emphasise passivity and diminished capabilities (92) and can contribute to more rapid decline (93) Older people in Western cultures can feel redundant and unvalued by society (94, 95)	Self-efficacy: confidence in one’s ability to cope with the challenges of dance. This confidence is carried into other aspects of life (96, 97) Pride in achievement: Taking pride in the accomplishment of learning new routines (skill attainment (89)) and putting them into practice (90, 98) Psychological safety: a positive, non-judgmental space where it is safe to be vulnerable and make mistakes (99) Defying expectations: pleasure in being an active older person who dances and defies stereotypical expectations (100–102) Feeling valued: experiencing positive reinforcement that your presence and contribution to the class matters and is welcomed (103) Together, these boosted self-esteem
Theory 3: RIPE Dance creates camaraderie	Use of a social pedagogy approach (104) informed by relational humanism (69, 105, 106) and ‘soft skills in dance’ (51) which focuses on encouraging and empowering facilitation Specific strategies for fostering camaraderie used by the teacher include knowing and using everyone’s name, introducing new participants conversationally, chatting with and demonstrating interest in all participants, caring enquiry about specific life events, sharing information with the group about events in participants’ lives (including celebrating birthdays), demonstrating caring through physical contact: (placing a hand on someone’s arm, an occasional hug), initiating optional social events beyond classes (coffee, lunches, performances), phoning people who have missed a class to see how they are, choreography that encourages interaction, and explicitly valuing participants’ support for each other in end-of-year ‘awards’.	Many older people have lost (or are losing) partners and friends, and experience decreased social contact (107). Social isolation and loneliness is associated with cardiovascular, autoimmune, neurocognitive and mental health problems (108) COVID-19 has exacerbated the growing ‘loneliness pandemic’ (109) Social connectivity is associated with appreciation of and adherence to exercise programs (59)	Social connection: feeling part of a group of friendly, like-minded people, a member of the RIPE Dance microcommunity (2, 90, 108) Mutual support: a sense of shared effort and intergroup support towards a shared goal (42, 110, 111) Rapport with the teacher: strongly positive feelings about the teachers’ skills, efforts, personality and interactive style, including wanting to ‘give something back’ (62, 112)
Theory 4: RIPE Dance is uplifting	Diverse music selection from “our era,” informed by a survey of participants’ favoured music in addition to other familiar or engaging music Exercise and dance routines incorporate synchronized movement that participants can ‘get right’ by the end of term Inventive choreography that often incorporates imagery, narratives, costume and props Sing-a-long dancing is encouraged (often led by the teacher) Teacher makes jokes, projects playful enthusiasm and leads laughter	Ageing is associated with poorer quality of life, e.g., anxiety and depression are common in older people (113). Those who are physically active have better self-efficacy, self-esteem and positive affect than inactive peers (114) Enjoyment is a key motivational driver for physical activity in older age (87, 89, 115) Dance may offer enhanced physical activity due to its incorporation of music and expressive, synchronized movement (2, 4)	Raised spirits: improved mood and wellbeing during and after classes (100, 115) Fun: engaging in laughter and playfulness (62, 89, 116) Synchrony: pleasure moving gracefully or rhythmically in unison with the music, the teacher and/or other class members (11, 36, 42) Musical reactivity: pleasure in dancing to (and singing along with) well-liked music (110, 117–119)

### 3.1 Program theory 1. RIPE dance benefits my body and mind

#### 3.1.1 Trust that the RIPE dance approach is ‘right for me’

Interviewees trusted in the safety and manageability of RIPE Dance classes. They were confident that classes were appropriately designed for older people and catered to different levels of ability. Safe, individualised practice was supported by continual guidance about working to one’s own level and followed two foundational principles: *“We have to be safe, and we have to have fun there. [The teacher] gave us those two rules, and she makes sure that that happens” (F67)*. Trust in the safety and appropriateness of the dance program was contingent on the teacher understanding and responding to each class members’ individual capabilities, something all interviewees remarked on.

#### 3.1.2 Belief in, and experience of, health benefits

Many interviewees explained that they anticipated health benefits from participating in the RIPE Dance program, hoping that the classes could *“give me a bit of an edge to age more gracefully, and positively” (F69)*. This belief was reinforced by the incorporation of educational comments by the teachers which made explicit connections between particular exercises and their physiological impacts. This boosted confidence that RIPE Dance classes are beneficial overall, but also that potentially less enjoyable exercises such as pliés (a commonly used knee bend in ballet) were worth the effort.

Self-reported health impacts included better leg strength, balance, mobility, flexibility, mood, cognitive dexterity, proprioception and sleep, and weight loss. Several people pointed out that they could not disentangle the effects of dance from the other physical activities they took part in, but assumed it was contributing positively to their health. A combination of cognitive, psychological, physiological and social benefits were often described. Overall, interviewees agreed that the RIPE Dance program was exercise in disguise.

Interviewees made a clear connection between physical activity and healthy ageing, seeing dance as a way to extend active living. They explained that classes pushed them to engage in exercise that targeted physical decline and helped them to *“feel [they] can do a lot of movements which [their] friends who do not attend the classes will not be able to do” (F69)*. Three interviewees with Parkinson’s disease were intentionally tackling their symptoms with these classes. A few interviewees also described improved physical literacy, especially listening to their bodies more effectively: “*I am learning more about what I can and cannot do” (F79)*.

All interviewees described the boost they felt from participating in RIPE Dance classes, but these impacts seemed particularly profound for those dealing with stress/anxiety, as well as for those experiencing low spirits caused by grief and/or social isolation. Stress was reduced by diversion from life’s concerns facilitated by multiple features of the classes including synchrony, musical reactivity, fun and laughter (theory 4), the flow of dance instruction and the necessity of focusing fully on dance. Some felt the benefits carried over into their everyday lives: “*I have a lot of things in my life that can be stressful…. it’s the dance that keeps me happy and keeps me getting through” (F66)*.

Several interviewees who had suffered significant losses found that RIPE Dance classes helped them deal with this pain. One woman who had struggled with bereavement explained how dancing provided time out from her grief, highlighting the beneficial role of focus, together with synchrony, laughter and group solidarity.

Nearly all interviewees emphasised the value of dance classes for their cognitive health: *“it’s protective for the brain” (F69)*. They described the mechanisms as challenges to memory and coordination which meant, *“the brain is engaged in problem solving” (F79)*. Several interviewees extended this challenge for greater cognitive benefits by trying to recall steps before they were called out, sometimes without watching the teacher.

### 3.2 Program theory 2. RIPE dance helps me feel better about myself

#### 3.2.1 Self-efficacy and pride in achievement

Interviewees found a great deal of pleasure in mastering challenging dance routines, emphasising the positive impacts on their self-esteem. Building confidence required genuine challenge so that, *“[You’re] achieving something that you did not think you were capable of” (F69)*. The pleasure this generated related to both cognitive and physiological achievement, and was noted even by those with sporting and/or dance backgrounds. Participants with Parkinson’s disease experienced increased capability in staying active and managing the disease. For some interviewees who lacked self-confidence in day-to-day life the development of greater dance-efficacy had repercussions beyond the classes: *“I feel more confident because I do it, and I feel more open to other challenges” (F79)*.

Those with no previous experience of dance often felt especially challenged in early classes where they were encountering entirely new ways of moving. Their accounts suggest that beliefs about health benefits were more important for this group who could not draw on experiential knowledge about the pleasures and benefits of dancing and took more time to develop self-efficacy.

Across all classes, self-efficacy was fostered via progressive routines where participants collectively learnt and built on previously mastered steps, with individualised support where needed. This was aided by the teachers’ expertise in gerontologically-attuned dance and their responsiveness to each participant. Closely related to self-efficacy was the pride of accomplishment from taking on these challenges and honing routines which they could usually perform smoothly by the end of each term: *“I’m very proud of the fact that my age, I’m still going to dance classes” (F84)*.

#### 3.2.2 Defying expectations

Interviewees talked about the challenges of ageing in an ageist society that stereotypes older people and talks down to them: *“I get all this “love” and “darling” from people who are younger. It drives me nuts. It’s so patronising!” (F79)*. They described pleasure and pride in countering these stereotypes and laughed about the surprise expressed when they tell people socially that they attend dance classes: *“[Telling people you dance] is a nice thing to do when people think you are a silly old lady” (F78)*. Defying expectations was pleasurable in itself, but also boosted self-esteem.

#### 3.2.3 Feeling valued

When explaining why they felt valued, interviewees described how the teachers showed appreciation for the effort each person made in simply turning up and then working hard in classes, and how teachers boosted participants’ self-esteem through continual positive reinforcement. This was underpinned by the friendly, interested and equitable way in which teachers related to class participants: *“they always take time to chat with you” (F71)*. The teachers’ interactive style was modelled and facilitated so successfully by the teachers that, across all classes, interviewees reported that they and the other participants interacted similarly.

#### 3.2.4 Psychological safety

Comfort with the challenge of RIPE Dance classes was supported by an environment which was *“totally non-threatening. You can mess up and it does not matter” (F83)*. Interviewees described shared laughter at class members’ frequent mistakes without any sense of self-consciousness because *“you are not on display. There’s no competition” (F75)*. This was modelled by the teachers in their supportive interactions and by explicitly reminding participants that mistakes are irrelevant as the aim is to enjoy themselves and have fun.

### 3.3 Program theory 3. RIPE dance creates camaraderie

#### 3.3.1 Social connection

Interviewees were extremely positive about the group dynamics of classes, describing a *“dance community” (F66)* of *“lovely” (F84), “friendly” (F89), “easy-going” (F75)* and *“like-minded” (F66)* people, generating *“a sense of belonging” (F74)*. This camaraderie was enjoyed by everyone, including self-described introverts and ‘social avoiders’, but feeling connection to the teacher and group members seemed to have greatest value for those who were experiencing social isolation or were socially restricted by caring responsibilities, *“We, like a lot of “older adults people, were really quite isolated. So I think the social aspect really does improve your mood, and that has to be good for your health”* (F83).

Many interviewees suggested that the homogeneous composition of the group, together with a shared purpose and mutual struggles, was an important aspect of this affinity. This related primarily to age, but some also highlighted the benefits of all-female classes; however, those who attended mixed-gender classes, including two male interviewees, were equally positive about the group dynamics.

When asked how these group bonds manifest, interviewees frequently gave examples of being warmly welcomed and of casual chat that included genuine social inquiry. But the process of simply dancing with others to music they all enjoyed (Theory 4), and celebrating the group’s achievements, also seemed to strengthen these bonds.

#### 3.3.2 Mutual support

A key component of what made these dance classes into microcommunities was a sense of shared caring and commitment to the group effort: *“we all look out for each other” (F89)* and *“egg each other on” (F83)*. There was an insistence on teamwork that may have been supported by the non-competitive class dynamics that teachers nurtured. For example, one woman with a musical background who said she sang along with the music was asked by the interviewer if she sang harmonies. She explained she chose not to because: *“I’m not there to push myself forward in any way. I’m there to actually be part of a team, to be part of a group that we are all doing the same thing. I’m not trying to be smarter than they are” (F79)*.

#### 3.3.3 Rapport with the teacher

Interviewees were universally enthusiastic about the skills and interactive styles of the teachers, describing these as essential to their enjoyment of classes. In particular, they described the *“positivity” (F79)*, *“empathy” (M81)* and *“encouragement” (F92)* of the teachers, and their ability to connect with every member of the class, making them feel valued and cared for as individuals. There were many accounts of teachers using inclusivity techniques to bring people together, including modelling caring concern for class members and interest in their lives, using names, sharing information and celebrating or commiserating about life events. Interviewees explained that the teachers interact with them as peers and are *“very much part of the group” (F83)*. They expressed considerable admiration for and appreciation of the teachers’ creative efforts in developing new engaging routines every term: *“Each time there’s something new and something interesting” (F74)*.

### 3.4 Program theory 4. RIPE dance is uplifting

#### 3.4.1 Raised spirits

The most prominent theme in interviewees’ accounts of engagement with RIPE Dance classes was enjoyment and the *“uplifting” (M81)* effect which caused participants to leave feeling *“bubblier” (F75)* with *“a zip in my step” (F69)*. Many found this elevation in mood was sustained after classes and suggested that it contributed to resilience, which enabled participants to manage life challenges more effectively.

When pressed to identify the mechanism(s) that lifted them up, participants talked about the skills and warmth of the teachers and group camaraderie (Theory 3), the joy of synchrony and connection to music, plus fun and spontaneous laughter (described below). They noted that the challenge of learning routines and putting them into practice in a safe space (Theory 2) provided distraction and *“…a really good escape” (F67)* from cares and stresses. Lifted spirits were also, in part, due to the immediate physical impacts of the classes: *“I feel really good and free and everything moves. My body moves totally. Oh, yes. It’s a happy feeling” (F79)*.

#### 3.4.2 Fun and laughter

The uplifting effect of the classes, and the frequent laughter they generated, was enhanced by the teachers’ sense of fun, cheeky use of props and costumes, and choreography that encouraged playfulness. There was strong appreciation of the teachers’ skills in facilitating *“silly stuff” (F69)*. Many interviewees also made jokey comments during classes, often cheerfully disparaging their dance skills.

Interviewees seemed to experience a kind of contagious positivity and enthusiasm, sparked by the teachers but reflected by group members. This created a heightened energy that was more pronounced with larger numbers of participants: *“I think it’s more fun with more people… we bounce off each other”(F75)*. But this emphasis on fun did not undermine the purposeful goal of exercise for healthy ageing and fall prevention: *“It’s serious, but it’s enjoyable at the same time” (F89)*.

#### 3.4.3 Synchrony

Nearly all interviewees described a deep satisfaction of moving in time with the rhythm of the music and, increasingly as each term progressed, being in sync with the teacher and classmates. Part of this pleasure related to *“the challenge of being in sync” (F79)* and to a sense of group unity when the music and everyone’s movements come together: “*I love feeling that I’m in tune with everybody else … that sense that we are all doing the same thing, we are all moving as one” (F74)*. The pleasure of synchrony, and of participants’ achievement in working as a group, was reinforced by the teachers verbally, and with the occasional video of an end-of-term performance. There was also pleasure in observing, and attempting to match, the graceful movement of the teachers.

#### 3.4.4 Musical reactivity

The pleasure of dance and of synchrony was strongly enhanced by the teachers’ use of *“lovely music” (F79)*. Teachers survey participants to identify their favourite songs and artists, then choreograph this music for classes. Thus, the music tended to be much loved and was frequently music that participants had known (and danced to) as teenagers and young adults. Connection to this music added to the fun of classes and was reinforced through singing along, but several interviewees described how it touched them profoundly, transporting them beyond mundane physical activity: “*Music gets to the soul in a way that I do not think if you are just riding a bike or running or doing something like that, it’s not the same… music seems to speak to me in a deeper way” (F74)*.

Music also supported the cognitive challenge of mastering complex dance routines. Teaching techniques maximised this via the use of narrative imagery which also served to heighten enjoyment, enhance aesthetic and expressive movement, and provide cognitive ‘stepping-stones’ through challenging routines. For example, in the class we observed, a repeated sit-stand-reach exercise was set to the song ‘Up, up and away (in my beautiful balloon)’ and facilitated by imagery related to the lyrics which embellished the movements within a larger ‘ballooning’ story, giving the sequence narrative meaning and coherence (Table 2).

**Table 2:** A selection of interviewee quotes illustrating the content and scope of RIPE dance program theories.

Mechanisms	Illustrative quotes
Program theory 1. RIPE Dance benefits my body and mind	
Trust that the RIPE Dance approach ‘is right for me’	*She always stresses that you work within your own level. You do not tax yourself beyond what you feel capable of doing. And it’s perfectly okay if she has suggested a particular movement, if one cannot quite execute it to perfection, it does not matter. So it’s a no-stress activity from the point of view of the teacher trying to draw the best out of each individual but without anyone feeling pressured about it. (F79)* *… she’s always aware if anybody’s going to have trouble…. she’ll get an extra chair for them to hold onto or something like that. She knows the people in the group who would have difficulty, for instance, sitting and standing without holding onto a chair…. She’s very aware about our disabilities. (F83)* *… she’s very, very well aware of our limitations. She knows full well that my left leg is the weaker of my two legs and so she does not try to push me into doing something beyond what I personally feel comfortable about doing. There’s nothing that [the teacher] gives us to do that I cannot do at 84. (F84)*
Belief in and/or experience of health benefits	*Physical health benefits* *[the teacher] explains to us quite often what particular moves and routines help us physically. So we are understanding that this will help with falls prevention or make you more agile or whatever. So she’s tailored it for the things that are good for people our age. And …she’ll tell us in depth what different things are actually helping us with. (F69)* *we get to do a lot of things that I would not do if I wasn’t at class. For example, standing on one leg and balancing and standing on tippy toes on two feet and doing the balance that way. And there’s a lot of other things like pliés, which I actually hate doing, but I know it’s good for the thigh muscles and everything. It’s all about strengthening the body so you are less likely to become frail and doddery…. I really enjoy it, and I know it’s good for me. (F74)* *I’m more and more aware of what has become, well, after years of sedentary activity… my back is drooping so I’m trying to stand and sit straighter and listen to my [body]. The knee, it’s not a painful protest, but it says, “Hey, slow down!” So that awareness of what’s happening has been enhanced in lots of ways. (F80)* *Psychological health benefits* *When you are dancing, you feel more invigorated and ready to go back to whatever it was. (F80)* *I am a much more open person and I am a much more happy person. (F69)* *if you have been a bit concerned about something, it can help free you of that because you are concentrating on something else, so you forget all about it. (F78)* *Grief is like your constant companion…. But when you are in your dance class, it’s …. not on your shoulders anymore because you are concentrating on learning a new dance routine and enjoying being with other women, and giggling, and laughing, and moving to music.. It’s hard to describe, but it’s the most wonderful thing…. More dance in your life helps with grief and all sorts of things. Mental health, physical health. I just love it… You come out on a high after dancing. And the moment you get to class, you feel great. You see all the other girls, you chat what you have been up to, and then dance classes, the music starts, you start doing your warmups, and it’s just the music and the movements. You start to feel good. And you are all there feeling the same thing, and it’s washing away the stresses of your life in the movement and the music, and just being part of a group of like-minded women. (F66)* *Cognitive health benefits* *… it’s also keeping the brain working because you have got to remember the steps…. We have to think what we are doing and we have to listen to the music so that we are putting our movements in with the music. So we are using lots of elements of our body, our mind, and our hearing. It’s fantastic. (F84)* *I’m trying to stop [Parkinson’s] progression and that’s what exercise does. And I’m trying to maintain my cognition, and that’s what dance helps with because I have to remember the moves…. Every now and again [the teacher] will say, “Who can remember what comes next?” And that’s like a challenge. So it’s about maintaining the best life that I can. (F67)*
Program theory 2. RIPE Dance helps me feel good about myself	
Self-efficacy	*the fact that you can cope with whatever we are learning to do and you are doing it well and you are doing it well in the company of other people who are also achieving, it must lift your spirits, must lift your confidence…. If you can cope with … those intricate steps … then you can cope with a lot of things that you did not expect that you could cope with. (F84)* *The ability to exercise, even if you are doing it at your own level, it does give you quite a lot of confidence. (F75)* *it’s given me a lot more confidence into how long I can keep going [despite Parkinson’s]. (M81)*
Pride in achievement	*it makes you feel proud of the fact that your body can move like it’s moving. (F84)* *When you nail a routine and you nail it without having to watch [the teacher], and you know you have learned it: wow! You feel like you have just mastered something huge and major. It’s only a little dance routine, but it puts you on a high. (F66)*
Defying expectations	*I think they are impressed that we ancient folk can still do things. (F83)* *I was … falling into line with what people expected me to do at this age. [Now] I just say to them, “I do not care what you think. It makes me feel happy, so I’m going to do it”. (F71)*
Feeling valued	*the fact that everybody is respected and valued makes you feel good…. You’re not ever made to feel like an idiot or not worthy or anything like that. And nobody puts anybody down…. And people are saying nice things…, “Oh, it’s great to see everybody here today. It’s good that you could come”. That sort of thing. So that makes you feel good about yourself as well as actually moving. (F67)* *… when I go in, I feel very welcome. I feel valued as a member of the class and everybody’s very friendly…. it’s just a lovely atmosphere…. And sometimes if I walk in late … [the teacher] will go, “Oh, Robyn’s here.” And everyone will go, “Yay!”. (F67)*
*Psychological safety*	*… there’s no judgement, there’s never any feeling that you have failed. You do what you can do, and that’s really good. While she’ll correct you up to a point, she never singles you out.…You come away with a feeling that, “I did that, I’m capable”. And that’s really important to one’s sense of wellbeing and self-esteem. (F74)* *I’m not the sort of person that can put myself in front of somebody and just start conversation. I cannot do that. But actually going to these dance classes, I can actually go in there and have a conversation with people that come in without having to wait for them to start it. (F71)*
Program theory 3. RIPE Dance creates camaraderie	
Social connection	*It’s meeting up with my fellow dancers once a week, and we have chat and that social connection, a sense of belonging to something. I think as human beings we all deep down crave connection with other human beings and the world we live in with all the screens and everything, connection becomes less and less …. But what you miss out on is the incidental exchanges…. That’s what I find really good about going there and meeting up with people. (F74)* *They make me feel welcome and at home, and that is a really big improvement for me. (F71)*
Mutual support	*We’re all there to help each other and enjoy what we can and cannot do, if you know what I mean. We’re all at different levels and we all enjoy what we can do, and have fun. It’s great…. We’re all sharing and helping each other, and following each other, and supporting each other. And no one’s singled out or targeted out. It’s very inclusive and sharing. (F66)* *There’s no competitiveness at all in the classes … and that’s something that [the teacher] fosters, the way that she interacts with everybody. There’s nobody that’s trying to be the best or anything like that, and everyone’s supportive of everybody. (F69)* *“…if we get it right, we congratulate each other…. So every now and again we pat each other on the back and go, “We did it! We did it!” (F67)*
Rapport with the teacher	*She radiates caring, she’s encouraging, she praises, she makes you feel good and she welcomes you. She’s so happy to see you each time you turn up. (F84)* *… if people do not turn up for a couple of weeks, [the teacher] will phone them and just check they are okay. Or if someone’s going away, she goes, “Now I know that this person’s not going to be here because they have gone on a cruise or something like that. (F67)* *She does a lot of things to encourage social interaction between the dancers by putting on regular morning teas when we get together, or a Christmas party …and she’ll have news of people in the class that are doing something else. She keeps in touch with everybody and she definitely puts a lot of effort into us getting on as a group of women…. Everyone’s birthday is always remembered. Anything that’s happening in someone’s life that needs to be talked about, that’s happy or sad…. And then afterwards a lot of people go and have coffee together…. And she actually does communicate that she is actively doing things to ensure social cohesion. She’ll give a little prize for people that are doing something unexpected. (F69)* *I think that’s pushed me to keep going because she puts in so much. I feel she puts in so much effort that I would not just drop out. (F75)*
Program theory 4. RIPE Dance is uplifting	
Raised spirits	*I suddenly realise I’ve got a big smile on my face. I think to myself, “God, I must look stupid.” I look around at some of the others and they have also got a big smile on their face…. Obviously, it makes us feel good. (F79)* *I feel really good when I come out, and then I come home and I’m actually more positive in what I’m doing around the house, and I sleep very well those days. (F71)* *It’s not an easy life at 84 that I’ve got. But knowing that I’ve got those joyful periods of dance to go to sort of uplifts you and things do not seem half as bad… I’m just a brighter person, I think. Lighter and brighter because I’ve got that [dance class]. I have an interesting break rather than seven days a week being a career. It’s lifted my spirits a lot…. It’s keeping me alive. (F84)*
Fun	*All the time we giggle and laugh. And we laugh at ourselves and each other. And no one’s made to feel inferior or above anybody. We’re equals. (F80)* *It’s just everybody there dancing together and laughing if you make a mistake, smiling, enjoying the music…. There’s a lot of laughter…. You sometimes think, “Ooh, dear. Fancy me doing this at my age! I’m supposed to be old and steadfast and whatever, but here I am acting like a teenager….” No, it’s all light-hearted and it’s fun and it’s doing me a lot of good. (F84)*
Synchrony	*There’s great pleasure to be had in being in sync with the others. (F80)* *[When] I’m at the back line watching all the other girls in front of me and we are doing a routine … and we are all in sync, it is absolutely wonderful. And I’ve often said when we finish, “Wow, girls. You all look fantastic!” … It is a wonderful feeling. (F66)*
Musical reactivity	*The music she plays is our era, [it is] the old music of our days. It brings back memories. We … relate to it because we know the songs…. And sometimes, quite often, we sing along with it as well because we know the words. (F89)* *You are almost not thinking what you are doing, you are just doing it because the music is telling you virtually that this is where you put your feet in. You’ve developed a lot of confidence in your own body and your mind…. enjoyment of music with others is a big part of it (F80)*

### 3.5 Unsupported mechanisms

Concepts of flow, embodiment and mindfulness were identified as potential mechanisms in our initial program theories and were thus explored in multiple early interviews. These interviewees did not allude to these concepts unprompted and were either lukewarm or, most often, dismissive of them when prompted. For example, they argued that they were concentrating too intently on ‘what comes next’ to be in a flow state:

I can’t say that I’ve really been in such a state there with this dance group that one gets totally lost in it. I think maybe if one was a well-trained and skilled ballet dancer, I could understand maybe mentally they’re becoming lost in it. But no, I wouldn’t say that that applies to us (F79).

And while this intense focus certainly kept them in the present moment, it did not produce the transcendence they tended to associate with mindfulness, *“It’s making my brain work in a different way than it does with other things that I do … but that’s about focus…. I just know I have to concentrate hard” (F69)*. While several alluded to improved interception they did not report relating to their bodies differently as a result of dance, or of personalised emotional expression through movement.

## 4 Discussion

Our four program theories and their 14 mechanisms were identified consistently in the interviews with 20 participants as providing the best explanations for their long-term and enthusiastic attendance at RIPE Dance classes: 1. RIPE Dance benefits my body and mind (trust in the RIPE approach and belief in health benefits), 2. RIPE Dance helps me feel good about myself (self-efficacy, pride in achievement, psychological safety, defying expectations and feeling valued), 3. RIPE Dance creates camaraderie (social connection, mutual support and rapport with the teacher), and 4. RIPE Dance is uplifting (raised spirits, fun, synchrony and musical reactivity). These findings align with factors identified as successfully helping older people maintain long-term adherence to community-based exercise programs including: the teachers’ caring encouragement and responsivity to individual needs; activity that is fun and can provide a form of escape; social interaction that fosters a sense of belonging; and perceptions of achievable activity that has physical and psycho-social benefits to support healthy ageing (62).

The enthusiasm of our interviewees echoes the views of older people in other studies of community-based dance classes in which they report feelings of “joy” and “euphoria” when dancing (26, 120), and positive existential emotions such as feeling alive, valued and part of a community (100). Building on sociological theory, Alfredsson-Olsson and colleagues (120, 121) explain this sense of group pleasure in dance as a highly successful interaction ritual. They argue that emotion is strongly shaped by one’s social engagement and that interactions can create a chain reaction that ‘transmits’ emotional energy throughout a group. This study identified multiple ways in which the RIPE Dance program facilitates highly successful interaction rituals. Some of this is simply leveraging natural characteristics of dance such as rhythmic and synchronized movement to music, which can intensify interaction rituals and generate collective effervescence (35). Singing along with the dance music and the frequent laughter observed in classes are both mood enhancing activities in their own right and likely to amplify positive emotional energy (110, 116, 122). Physical synchrony, group singing and shared laughter are all strongly associated with social bonding (39, 41, 110, 116). However, the RIPE Dance approach seems especially effective at combining these elements to produce highly successful interaction rituals. This may be explained by the teaching methodology which is strongly informed by ‘soft skills in dance’, focusing on empowering and community-building facilitation (51) and a relational humanist approach (69, 105, 106). This holistic ‘heads, hearts and hands’ pedagogy can optimise cognitive, affective and psychomotor learning (123) and has been used in other programs for older people that employ creative approaches (104).

Next, we focus on four specific strategies RIPE Dance teachers use to facilitate this highly engaging dynamic with an emphasis on transferable lessons for other dance classes.

### 4.1 Meaningful music

The strategy of teachers selecting engaging music, including music “from our era” in consultation with the group, was highly valued by interviewees and considered to be a key element in their enjoyment of dance classes, including their sense of inclusion and validation. Neuroscience shows that listening to a favourite song alters the connectivity between auditory brain areas and the hippocampus, a region responsible for memory and social emotion consolidation (124). People with dementia seem to respond better to music they grew up listening to Sugaya and Yonetani (125), while enhanced familiarity with music has been found to improve stride amplitude and variability and to reduce cognitive demand in people with Parkinson’s (117).

Interviewees frequently alluded to the power of music in RIPE Dance classes to move them physically and emotionally; a well-established phenomenon in the literature (118). This may be in part because of its ability to tap into an ‘architecture of memories’ from the past that can elicit deep emotional responses (126). This shared enjoyment of music also strengthens social bonding and, as our interviewees suggested, can generate group energy which is strengthened when greater numbers of people take part (118). These intragroup bonds are especially significant for adherence as social bonds formed during shared physical activity can strengthen perceived obligation towards the group and thus provide motivation to sustain group membership (88).

### 4.2 Narrative and imagery

Interviewees also enjoyed the teachers’ playful use of narrative and imagery in their instruction, sometimes involving props. While they focused on the fun this evoked, the literature suggests the impacts may be more profound. Imagery in dance instruction can aid movement precision and provide mnemotechnic stepping-stones for recalling routines, but it also creates a fantasy element that can tap into aesthetic and emotional expression and enjoyment (33) and facilitate escape into another world (112). It is also likely to enhance the therapeutic properties of mind–body connection noted in studies of imagery in Tai Chi (127). These features can contribute to psychological restoration that *“promotes recovery from a depleted resource such as cognitive fatigue, stress, or low mood”* [100:486]. This may explain why engagement in creative and cultural activities is a primary contributor to wellbeing in later life (4, 104, 119). Christensen et al. argue that recreational dance teachers should use imagery whenever possible to enhance both movement and enjoyment (33). The use of imagery combined with observation of the teacher modelling movements may have greater benefits (56).

### 4.3 Making dance accessible for all

The teachers using the RIPE Dance approach constantly reinforced the message that dance *really is possible for everyone* and used practical strategies to support this. They offered individualised guidance about which of their varied ability classes are best suited to each person, suggested modifications for people with chronic or acute conditions, and used chairs creatively to create higher seated positions, balance aids and a ‘corridor’ for those with pronounced mobility limitations. Importantly, the RIPE Dance approach provides graduated challenge across each term, and from term to term. Devereux et al. (88, 89) point out that older people prefer incrementally increasing challenge that gradually raises self-efficacy. This ‘scaffolds’ the power of dance to promote self-esteem and self-efficacy which can extend to other aspects of life (7, 37, 128), and counters deficit discourses of ageing which can contribute to negative psychological wellbeing ad cognitive decline in older people (93, 129). Our interviewees’ accounts of “defying expectations” align with research that found older people used dance to resist ageist stereotypes (100). Frequent positive comments made by interviewees about older members of their dance class also suggest empowering peer role modelling of active ageing (130). This is especially important as more positive expectations of ageing are associated with higher ongoing engagement in physical activity (90) as well as improved psychological wellbeing (129).

The teachers also demonstrated accessible forms of dance in the community more broadly. As this interviewee explained, she only attended RIPE Dance classes because one of these demonstrations completely changed her belief she could not dance:

… seeing the way that [the teacher] did the dance in the nursing home made me realise that dance was more than ballet. It could be anything. It could be just moving your eyelids in time to the music. And so that made me realise that this was a mindset thing, that I could dance to the extent that I was capable (F74).

Such strategies for building confidence pre-program may be crucial because dance efficacy has been found to be weaker in populations who could benefit most from programs like RIPE Dance such as older people and those with poorer mental health, cognitive and physical abilities, physical activity levels and social networks, and because poor dance efficacy before starting a dance program is associated with lower attendance overall (131).

### 4.4 Facilitating a humanising environment

The RIPE Dance approach strives to combat social alienation and loneliness by facilitating a warmly welcoming environment in which individuals feel valued and are encouraged to feel comfortable, laugh, be playful and mingle. Thus teachers using the RIPE Dance approach act as both dance instructors and social coordinators (132), drawing on a social pedagogy to build community capital and promote wellbeing, learning and growth. Hunter argues that this approach can help older people to reclaim their sense of self, self-confidence and joy in human connection, and *“is precisely what is needed to counter the hegemonic belief that older people’s wellbeing is of little consequence”* (104).

Despite the reputation of dance as an activity that increases self-consciousness, we found teachers using the RIPE Dance approach had created a non-judgemental group space in which participants felt a sense of belonging and were content to dance according to their own capabilities. This sense of psychological safety can support physical learning and performance competencies, self-efficacy, self-worth and positive social connections (99). Camlin reports similar themes in relation to group singing and extends the concept of psychological safety to the healing experience of feeling connected to others without intense intimacy which can feel overwhelming (110).

Killingback describes this as a *“humanising environment”* which supports sustained participation and should underpin the design of physical activity programs for older people (133). Others emphasise the importance of social connectivity in particular, and call for opportunities for social interaction to be embedded in the structure of group activities beyond that which may occur incidentally (134). This is important for combatting loneliness which is negatively associated with cardiovascular and autoimmune conditions, mental health, cognitive function, risk of dementia (135) and coping self-efficacy (31). Our interviewees were clear that RIPE Dance classes were uplifting and validating. This highlights the potential role of dance classes such as these in countering *“tiredness of life”* which is a growing trend in Western society, compounded by existential loneliness, physical decline, loss and sense of social redundancy (94).

### 4.5 Implications for scalability

Dance classes using the RIPE Dance approach have the potential to be scaled for greater access in the community and in aged care. A pilot randomised study testing a model similar to RIPE Dance which integrates physical therapy exercises for fall prevention with dance movement therapy has demonstrated the program’s feasibility and potential benefits (136). Dance classes require little equipment and are not resource intensive to organize, however they do require an excellent and committed teacher with technical, creative and interpersonal skills and a caring, outgoing personality (96, 112). Some gerontological and condition-specific knowledge is needed so that movements can be modified safely for different needs.

The form of dance is likely to be a vital consideration. Previous studies have shown limited or negligible benefits from social ballroom dancing classes on cognitive function (137). The only RCT to examine the impact of dance on falls in older people found no significant benefits of folk and ballroom styles, and called for investigation of a more structured fall-prevention focused program (138). The RIPE Dance program could provide this opportunity as it targets fall risks by incorporating progressive evidence-based exercises for leg strength and improved balance, and intentional cognitive challenge.

The question of frequency and duration of classes is also important. Studies suggest that even a low ‘dose’ of dance can have benefits. For example, a 6-month dance program for older people of 1 h per week was found to improve motor, tactile and postural performance and reaction time, and to enhance subjective wellbeing and cardio-respiratory performance, especially benefiting those with the lowest performance pre-intervention (5). Granacher et al. (139) suggest the program should exceed 8 weeks, while Merom et al. (138) argue that, if the aim is to prevent falls, programs should facilitate approximately 56 h of dancing (accrued weekly over a long period, or more intensively for a shorter duration) that includes high balance challenging exercises. For people with Parkinson’s disease, program duration of 12+ weeks (60 or 90 min per class) may be optimal (140). Shorter durations may be effective if mental health outcomes are the focus. A one-off dance intervention significantly reduced depression in psychiatric patients (29), and a mere 5 min of dancing was found to enhance positive affect, decrease negative affect and reduce feelings of fatigue in younger people (28), but it is not known if this would translate to an older population. Our study did not explore potential variations in benefits of attending RIPE Dance classes over different durations, but our interviewees were adamant that they wanted to continue to attend classes for as long as they were able. Most attended only once per week yet identified numerous benefits. Thus long-term programs offering one or two classes per week may align with the preferences of older people. The intensity of exertion may also be an important consideration as the World Health Organization guidelines on physical activity and sedentary behaviour recommend that all adults should undertake 150–300 min of moderate-intensity, or 75–150 min of vigorous-intensity physical activity, or some equivalent combination of moderate-intensity and vigorous-intensity aerobic physical activity, per week (141). To encourage uptake by older people who may feel daunted by these challenges, the promotion of dance programs should emphasise the benefits of dance for positive ageing and reinforce the RIPE mantra that dance *really is possible for everyone* (90).

### 4.6 What’s next?

Gold Moves Australia directors (the teachers who designed and deliver the RIPE Dance program) have developed and are implementing a training program in dance facilitation for health and wellbeing in older people based on the RIPE Dance approach. This includes developing skills and knowledge in adapting and modifying dance content, gerontological attunement, relational interaction and the understanding and competencies needed to create an environment that supports the program theories and activates the mechanisms identified in this study. Waiting lists, and the anxiety expressed in interviews about potential loss of RIPE Dance classes if the current teachers should retire or become ill, suggest that expansion of the RIPE Dance program would be highly valued. As one of our interviewees put it:

… the most important thing, the end outcome, is that there are more classes like this available for people to attend. Because from my knowledge of physical things in ageing, it’s the best thing that I’ve ever come across. Other exercises are excellent, but there are many extra benefits to dancing (F69).

### 4.7 Strengths and limitations

The trustworthiness of the program theories developed in this study was strengthened via the realist technique of iterative testing and refinement in interviews, and interrogation of initial program theories by the research partnership team, plus triangulation of interview and observational data (78, 142–144). However, these findings cannot be applied indiscriminately to wider populations of older people. We only interviewed current dance class attendees, all of whom had completed a minimum of 8 months dancing and had returned for at least one subsequent term. So this was a self-selecting group who had chosen to enrol in dance classes and were sufficiently engaged to continue to participate. There was moderate socioeconomic variation as determined by postcodes, but little cultural and linguistic diversity. Realist evaluation contends that no program works for everyone in all circumstances (145) so meeting the needs and preferences of minority and disadvantaged groups would require specific investigation. The two interviewees who spoke English as a second language said that understanding verbal instruction was less important in a class where movement is modelled continuously, however people may be more attracted by dance (and music) that has personal (and thus cultural) resonance (146). The principles underlying the RIPE Dance approach would support such adaptation. For example, Gold Moves Australia proposes that teachers understand and reflect the demographic profile they are trying to reach, use highly relational and responsive teaching strategies, select music that connects with and engages attendees and devise dance sequences with some familiar, meaningful movement. These features mean that the RIPE Dance approach is well placed to work for different sub-populations of older adults.

## 5 Conclusion

Our realist evaluation found that the RIPE Dance program provides challenging and hugely enjoyable exercise in disguise for older people with a range of mobility profiles. It is well established that engagement in creative and cultural activities is a primary contributor to wellbeing in later life (4, 104, 119), but dance may offer a unique contribution (33, 147). Participation in RIPE Dance classes generated multiple, synergistic effects: benefits to body and mind, impacts on self-regard, group camaraderie and a sense of being uplifted. Physical activity has long been regarded as a ‘polypill’ with the capability to enhance physical, mental and cognitive health and quality of life (148). Building on this concept, we suggest that dance programs, especially those like RIPE Dance which use established and evidence-based techniques to target physical, psychological, cognitive and social wellbeing, may function as a ‘plethopill’,^1^ activating myriad mechanisms that can engage a wide range of older people long-term and contribute significantly to their experiences of healthy, happy ageing.

## Data availability statement

The datasets presented in this article are not readily available because they contain identifiable participant information. Parts of the dataset that can be fully deidentified and will not compromise the anonymity of participants can be made available upon request. Requests to access the datasets should be directed to AH, abby.haynes@sydney.edu.au.

## Ethics statement

The studies involving humans were approved by the University of Sydney’s Human Research Ethics Committee, study reference 2022/772. The studies were conducted in accordance with the local legislation and institutional requirements. The participants provided their written informed consent to participate in this study. Written informed consent was obtained from the individual(s) for the publication of any potentially identifiable images or data included in this article.

## Author contributions

AH: Conceptualization, Data curation, Formal analysis, Methodology, Writing – original draft. AT: Conceptualization, Formal analysis, Supervision, Writing – review & editing. GH: Formal analysis, Project administration, Writing – review & editing. JC: Formal analysis, Project administration, Writing – review & editing. CS: Formal analysis, Writing – review & editing. DM: Writing – review & editing. HG: Conceptualization, Formal analysis, Funding acquisition, Investigation, Project administration, Writing – review & editing.

## Footnotes

^1^Poly comes from the Greek polus to mean more than one or many, whereas pletho comes from the Greek pléthos to mean a great number or multitude.
