# Supplementary material for: “It doesn’t feel like exercise”: a realist process evaluation of factors that support long-term attendance at dance classes designed for healthy ageing
Source: Front Public Health. 2023 Dec 20;11:1284272. doi: 10.3389/fpubh.2023.1284272 (PMC10773813; doi:10.3389/fpubh.2023.1284272)
Supplement: Supplementary file 1 [file Data_Sheet_1.PDF]

Research Study: Is dance exercise in disguise?

INTERVIEW GUIDE

Thank you for agreeing to take part in this interview. As you know, we're evaluating the dance classes that you've been taking part in. We hope that learning more about your experience will help us (and other researchers) design and promote programs like this more widely.

Before we start, I want to let you that I'd like to **audio record** our conversation so that I capture your views accurately. But I can reassure you that our conversation is confidential which means that when we talk about or publish our results we won't use any information that reveals your identity. Does that sound OK?

Also, can I please double-check that you understand the purpose of this interview and you're **happy to take part**? Great – thanks!

Part A. Reasons for participation

1. Thinking back to when you first heard about the (RIPE) dance classes, what made you decide to take part? What were you hoping to get out of it?

2. What were your views about dance at the time? Had you any previous experience with dance?

3. What about falls – was that something you were thinking about at the time?

Part B. Experiences of participation

4. We're keen to learn as much as possible these classes. What's your experience of them? Is there anything you would change/improve?

5. Life is complicated and it can be hard to stick with an exercise program. What helps you stick with RIPE Dance?

6. Is there anything that gets in the way or makes it difficult for you take part?

> Prompts: social, physical, mental benefits, improved function, enjoyment, quality of teaching, music, cost, location?

7. When classes moved online due to COVID, did you participate in those? If so, how would you compare the experience of learning dancing live to online?

8. Do you talk to family and friends about doing RIPE dance classes? What does it feel like to tell people you're a dancer? What do they say?

Part C. Impacts and maintenance

9. Do you think anything has changed physically or mentally for you because of taking part in these dance classes? Has it changed the way you think about yourself as a older person?

10. What about preventing falls - has that changed in any way since you've been doing the classes?

11. Thinking about the future now… Do you see yourself keeping up with dancing? What would help?

Part D. Theory testing

12. Part of our research into these classes is to develop a theory about what makes them work well for people who attend regularly. At this stage the theory is very rough ideas but we're using insights from interviewees (like you) to refine it. Can I tell you about some of the ideas in our theory and get your feedback on them?

**Outline theory**	**Some possible mechanisms - use as prompts in the interview**
RIPE/Come dance benefits my body and mind	• Confidence that RIPE dance is safe / tailored / age appropriate<br>• Anticipating and/or experiencing health benefits from such as better strength, balance, physical literacy, posture, flexibility, mobility, mental health (e.g. stress reduction), cognitive health and sleep<br>• A sense of wellbeing during and after classes
RIPE/Come dance helps me feel good about myself	• Pride in achievement [mastery]: learning new routines and putting them into practice (and overcoming self-consciousness?)<br>• Confidence [self efficacy] in one's ability to cope with the challenges of dance (aided by the balance of challenging new routines with comfort in progressive practice which builds on previously mastered steps). This confidence is carried into other aspects of life<br>• Sense of purpose in taking responsibility for looking after oneself<br>• Pleasure in being 'someone who dances', including being an active older person who is defying expectations
RIPE/Come dance creates camaraderie	• Feeling part of a group of friendly, like-minded people [social bonding]<br>• Feeling cared for and encouraged by the instructor<br>• A sense of commitment to the instructor – wanting to make an effort and give something back<br>• A sense of commitment to others in the group - a sense of shared effort and mutual support
RIPE/Come dance is enjoyable	• Dancing to (the right sort of) music feels joyful: pleasure in graceful or rhythmic movement and being in sync with the music and others in the class [synchrony]<br>• It is fun: there is laughter and playfulness (e.g. dances with costume or props)<br>• It is a positive space where it feels safe to be vulnerable and make mistakes<br>• Distraction from life's concerns (time out in a happy environment)

Prompt: Does that ring true for you, or maybe your experience is different? Can you give me an example? Do you think we've missed anything important in understanding the appeal of RIPE?

PART E. Final thoughts

13. Is there anything else you can tell us that might help us to understand how these classes work for people who attend them?

Thank you so much for helping us with our research. It's been really helpful to hear about your experience.
