# Supplementary material for: “It doesn’t feel like exercise”: a realist process evaluation of factors that support long-term attendance at dance classes designed for healthy ageing
Source: Front Public Health. 2023 Dec 20;11:1284272. doi: 10.3389/fpubh.2023.1284272 (PMC10773813; doi:10.3389/fpubh.2023.1284272)
Supplement: Supplementary file 2 [file Data_Sheet_2.PDF]

Theories	Possible mechanisms and observable data
**RIPE/Come dance benefits my body and mind**	• **Trust that the RIPE/Come Dance model 'is right for me'**: belief that it is safe, age appropriate and is individually tailored for each person's needs<br>• **Belief in and/or experience of health benefits** (physical, psychological and cognitive)<br><br>**Observation**<br>- Integration of techniques used to maximise strength, flexibility and balance in older people<br>- How different levels of ability/pain are accommodated<br>- Level of ability of participants<br>- Observable engagement during and after class (i.e. as participants leave)<br>- Any additional teaching strategies we have not picked up on in interviews
**RIPE/Come dance helps me feel good about myself**	• **Self-efficacy:** confidence in one's ability to cope with the challenges of dance.<br>• **Pride in achievement:** Taking pride in the accomplishment of learning new routines and putting them into practice<br>• **Defying expectations:** pleasure in being an active older person who dances and defies stereotypical expectations<br>• **Feeling valued:** experiencing positive reinforcement that your presence and contribution to the class matters and is welcomed<br>• **Psychological safety**: a positive, non-judgmental space where it is safe to be vulnerable and make mistakes.<br><br>**Observation**<br>- degree of physical self-confidence expressed by participants<br>- lack of self-consciousness, particularly when mistakes are made<br>- Strategies used by teacher to promote self-esteem and psychological safety
**RIPE/Come dance creates camaraderie**	• **Social connection**: feeling part of a group of friendly, like-minded people, a member of the RIPE/Dance microcommunity<br>• **Mutual support**: a sense of shared effort and intergroup support towards a shared goa<br>• **Rapport with the teacher:** strongly positive feelings about the teachers' skills, efforts, personality and interactive style, including wanting to 'give something back'<br><br>**Observation**<br>- Interactions between class members (and teaching strategies used to support this)<br>- Interactions with teacher<br>- Ethos of care brought to class by the teacher<br>- Leadership/commitment shown by class members
**RIPE/Come dance is uplifting**	• **Raised spirits:** improved mood and wellbeing during and after classes<br>• **Fun**: engaging in laughter and playfulness<br>• **Synchrony**: pleasure moving gracefully or rhythmically in sync with the music, the teacher and other class members<br>• **Musical reactivity:** pleasure in dancing to (and singing along with) well-liked music<br><br>**Observation**<br>- Enjoyment, fun expressed by participants<br>- Use of music/choreography and reactions to this
